# Myopia of Prematurity: Reduced Progression Using Highly Aspherical Lenslet Target (HALT) Technology

**DOI:** 10.3390/jcm15020484

**Published:** 2026-01-08

**Authors:** Raffaele Parrozzani, Carolina Molin, Alessandro Carli, Eleonora Cosmo, Evelyn Longhin, Giulia Midena, Edoardo Midena

**Affiliations:** 1 Department of Neuroscience-Ophthalmology, University of Padova, 35128 Padova, Italy; carolina.molin@studenti.unipd.it (C.M.); alessandrocarli27@gmail.com (A.C.); eleonora.cosmo@unipd.it (E.C.); evelyn.longhin@unipd.it (E.L.); edoardo.midena@unipd.it (E.M.); 2 IRCCS-Fondazione Bietti, 00198 Rome, Italy; giulia.midena@fondazionebietti.it

**Keywords:** myopia, myopia progression, preterm infants, retinopathy of prematurity, Highly Aspherical Lenslet Target (HALT)

## Abstract

**Objectives**: Myopia of prematurity (MOP) is a refractive error occurring in individuals born prematurely and is considered a distinct entity from pathologic and school-age myopia. Children affected by MOP are at risk of developing high myopia, with an increased lifelong cumulative risk of related complications. The aim of this study was to evaluate the progression of MOP in children previously affected by retinopathy of prematurity (ROP) who wore spectacles with Highly Aspherical Lenslet Target (HALT) technology compared to conventional single-vision lenses during childhood. **Methods**: Enrolled subjects were divided into two groups: subjects who used HALT lenses for at least 12 months and children who used standard single-vision lenses for the same period. The temporal evolution of spherical equivalent (SE) and axial length (AL) was evaluated in both groups. **Results**: Of the 252 preterm children screened, 58 were included in the study: 38 subjects (66%) in the standard lenses group and 20 subjects (34%) in the HALT lenses group. At 12 months SE progression and AL elongation in the HALT group (−0.32 ± 0.20 D and 0.12 ± 0.05 mm) were lower compared to the standard group (−0.93 ± 0.34 D and 0.46 ± 0.09 mm, *p* < 0.0001). **Conclusions**: The progression of MOP appears to be reduced in subjects corrected with HALT lenses compared to those wearing conventional lenses. These results suggest further investigation of HALT technology in selected subgroups of patients at high-risk of severe myopia to reduce its progression and the related lifelong cumulative risk of visual impairment.

## 1. Introduction

Myopia of prematurity (MOP) is a significant ocular complication that develops throughout the lifetime of individuals born prematurely, particularly those with a history of retinopathy of prematurity (ROP) [1,2]. With the progressive improvement in neonatal intensive care and the consequent increase in survival rates of preterm infants, the prevalence of MOP has also risen, making it a growing concern in pediatric ophthalmology [3,4]. Several studies have reported that the incidence of myopia in preterm infants is inversely associated with birth weight (BW) ≤ 2500 g and gestational age (GA) ≤ 30 weeks. Conversely, MOP is directly associated with the severity of ROP [5,6]. Additionally, the development and progression of myopia in children with ROP are increased in those who underwent treatments for type 1 ROP, such as laser photocoagulation or cryotherapy. In contrast, children with a history of type 1 ROP treated with intravitreal injections of vascular endothelial growth factor (VEGF) inhibitors show a less pronounced myopic shift [7,8,9,10]. Children affected by MOP are at risk of developing high myopia (≥−6.00 D) during their growth, along with progressive axial elongation of the eye, which in turn predisposes to an increased risk of ocular complications such as retinal detachment, glaucoma and early cataract [11]. Therefore, it is crucial to introduce interventions to manage and potentially reduce the progression of MOP.

The mechanism underlying the pathogenesis of MOP is still unclear and cannot be fully explained solely by increased axial length (AL), which typically elongate as the child grows (as observed in pathologic and school-age myopia) [12,13]. The disruption of the normal emmetropization process in preterm infants is multifactorial and partly attributable to arrested development of the ocular anterior segment after birth, resulting in a steeper corneal curvature, increased lens thickness and shallower anterior chamber depth. Each of these alterations, alone or in combination, contributes to the development of MOP in the first months of life [14,15]. Nevertheless, MOP progression during childhood, which is similar to that observed in pathologic and school-age myopia, also plays a significant role in determining the final refractive outcome of these patients. Therefore, slowing this progression may reduce the high prevalence of severe myopia in this high-risk population.

Recent advances in corrective lens design have sparked interest in reducing the progression of school-age myopia, including Highly Aspherical Lenslet Target (HALT) technology, which is based on highly aspheric lenslets distributed across the entire lens surface and aims to alter the distribution of peripheral defocus, considered a key factor in myopia progression. HALT lenses are characterized by a central clear zone for distance vision, surrounded by 11 concentric rings of 1021 tiny, invisible lenslets that create a “volume of myopic defocus” [16]. Studies on HALT lenses have demonstrated their potential to slow myopia progression in children affected by school-age myopia [17,18,19,20]. These findings suggested us to analyze the efficacy of this technology in selected subjects at high risk of severe myopia: children born prematurely that develop MOP precociously. Evaluating the effectiveness of such interventions may be crucial, as reducing MOP progression could decrease the lifelong cumulative risk of visual impairment related to myopia complications, ultimately improving the quality of life of these individuals.

The aim of this study was to evaluate the progression of MOP in children born prematurely and previously affected by ROP who wore spectacles with HALT technology during childhood, compared with those corrected with conventional single-vision spectacle lenses.

## 2. Materials and Methods

### 2.1. Study Population

This was a single-center, longitudinal, retrospective study compliant with the tenets of the Declaration of Helsinki and approved by the local Institutional Review Board (No. AOP3502_CTR). Informed consent was obtained from the legal guardian of each infant. The medical records of a total of 252 children born prematurely and previously affected by ROP, who were followed at the University Hospital of Padova between January 2022 and December 2023, were screened for patient inclusion. Exclusion criteria were as follows: children treated with laser photocoagulation for ROP, subjects with refractive data available for less than 12 months and subjects with refractive errors greater than −6.00 D of myopia or 2.00 D of astigmatism after cycloplegic autorefraction. Subjects with best-corrected visual acuity (BCVA) below age-based norms were also excluded. Enrolled subjects were divided into two groups: those who wore spectacle lenses with HALT technology for at least 12 months (HALT group) and those who wore conventional single-vision lenses for the same period (standard group). The following clinical characteristics were collected: GA at birth, BW, ROP type, maximum ROP stage (defined according to the International Classification of Retinopathy of Prematurity revisited [21]), need for treatment, type of treatment and age at glasses prescription. To facilitate the comparison among children with different ROP outcomes, our population was classified into two subsets, according to the ET-ROP study [22]: “type 1 ROP” included infants with any ROP requiring treatment, such as type 1 ROP and aggressive posterior ROP; conversely, “type 2 ROP” included preterm babies with type 2 ROP and mild ROPs, which spontaneously regressed. Spherical equivalent (SE) refractive error and AL were measured in both eyes at baseline, 6 months and 12 months. SE was obtained from cycloplegic autorefraction, using Tonoref III autorefractometer (Nidek, Gamagori, Japan), performed 30 to 45 min after Cyclopentolate 1% eyedrops instillation (2 administrations 5 min apart). AL was measured with IOL Master 500 optical biometer (Carl Zeiss Meditec, Jena, Germany) after the instillation of cycloplegic agent. Adherence to treatment was assessed using the self-reported average number of days per week of lens wear, as recorded in our standard clinical documentation (low when <3/7; moderate when >3/7; high when >6/7). Side effects were collected using our standard clinical data form as part of routine clinical assessments.

### 2.2. Statistical Analysis

All variables were evaluated using standard descriptive statistical methods: mean and standard deviation for quantitative variables, absolute and relative (percentage) frequencies for qualitative variables. The normal distribution of parameters was checked by the Shapiro–Wilk test. The evaluation of the temporal evolution of SE and AL was compared among both groups in each of the three timepoints by means of repeated measures analysis of variance (ANOVA). Comparison of SE changes between groups ware also performed using a multiple logistic regression model adjusted for GA, BW, ROP type, ROP treatment and age at glasses prescription. Data were analyzed using SAS^®^ v.9.4 statistical software (SAS Institute, Cary, NC, USA). A *p*-value < 0.05 was considered statistically significant.

## 3. Results

### 3.1. Population Features

Fifty-eight infants (23%) of the 252 preterm children screened fulfilled the inclusion criteria and were finally enrolled. The reasons for patient exclusion were as follows: laser photocoagulation for ROP (22 cases; 11%); availability of refractive data for less than 12 months (137 cases; 71%); refractive errors greater than −6.00 D of myopia or 2.00 D of astigmatism after cycloplegic autorefraction (28 cases; 14%); BCVA below age-based norms (7 cases; 4%). Of the 58 enrolled infants, 38 subjects (66%) were included in the standard lenses group and 20 subjects (34%) in the HALT lenses group. Participants contributed with both eyes to the study (116 eyes). The mean age at glasses prescription was 6.3 ± 0.8 years in the single-vision group and 6.2 ± 0.8 years in the HALT group. No statistically significant differences were observed between the two groups in age at glasses prescription, sex, GA, BW, ROP type, maximum ROP stage and number of subjects that needed ROP treatment (*p* > 0.05). Adherence to corrective lens wear was high (>6 days/week) in 36/38 standard lenses group patients and in 19/20 HALT group patients (*p* > 0.05). No complaints, such as blur, dizziness, headache and other symptoms or side effects were reported in both groups. Detailed clinical and demographics characteristics of the included population are reported in Table 1.

### 3.2. Refractive and Biometric Parameters

**Figure 1:**
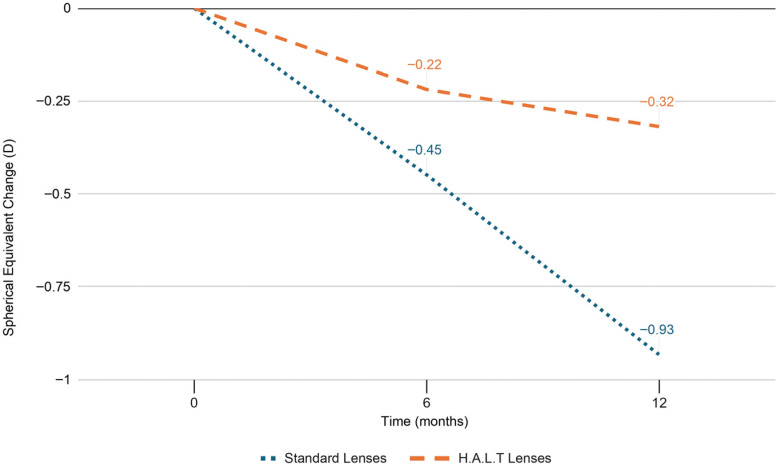
SE progression in standard and HALT groups at baseline, 6 and 12 months.

**Figure 2:**
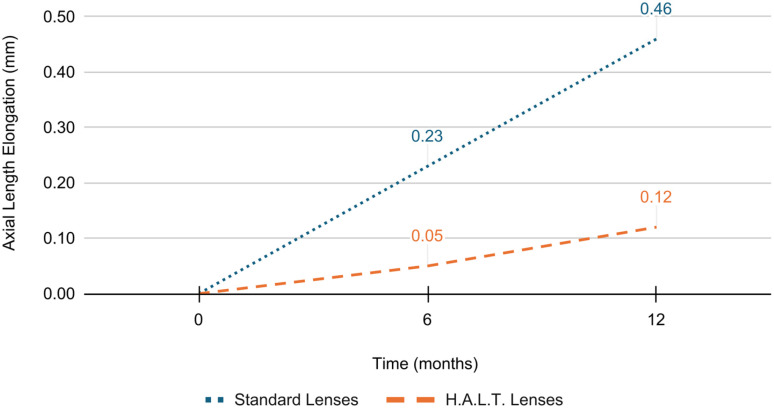
AL elongation in standard and HALT groups at baseline, 6 and 12 months.

At baseline, SE refractive error and AL did not significantly differ between the two groups (*p* = 0.846 and *p* = 0.840, respectively). At 6 months, SE progression was −0.45 ± 0.26 D and AL elongation was 0.23 ± 0.04 mm in the standard lenses group versus −0.22 ± 0.17 D and 0.05 ± 0.02 mm in the HALT group (*p* < 0.0001). At 12 months, myopia progressed by −0.93 ± 0.34 D and AL elongated by 0.46 ± 0.09 mm in the standard lenses group versus −0.32 ± 0.20 D and 0.12 ± 0.05 mm in the HALT group (*p* < 0.0001), as reported in Figure 1 and Figure 2. During follow-up, SE and AL mean values were significantly lower at both timepoints in the HALT group compared to the standard lenses group. Differences in SE changes and AL elongation in the two groups at baseline, 6 and 12 months are reported in Table 2 and Table 3.

### 3.3. Relationships Between Refractive and Clinical Parameters

**Figure 3:**
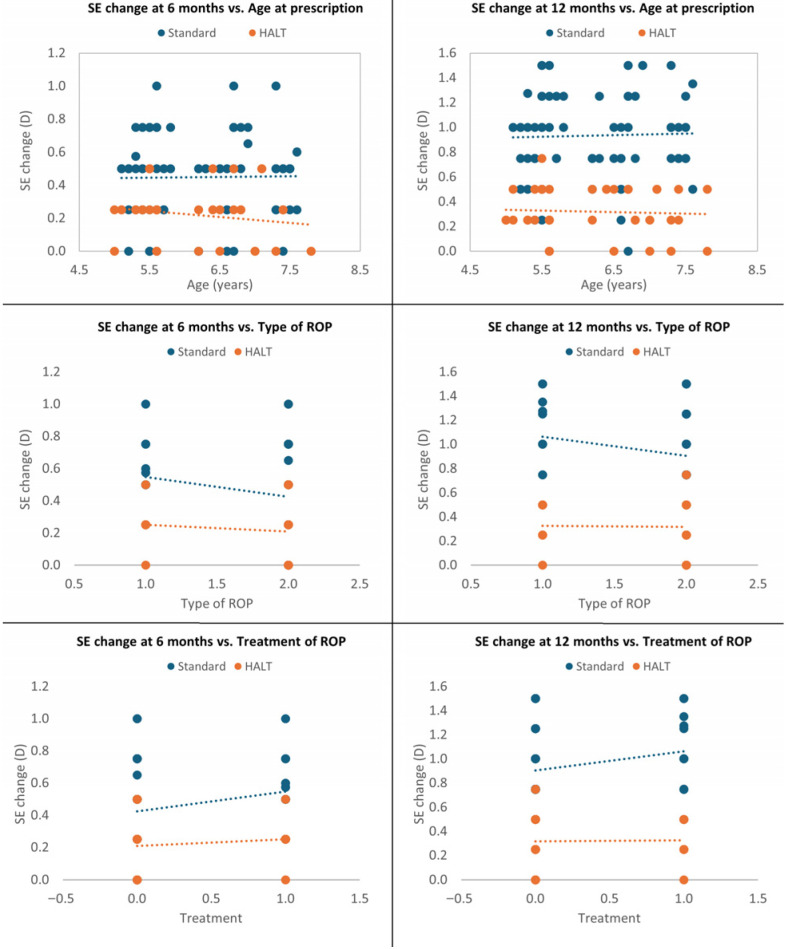
Multivariate analysis at 6 and 12 months adjusted for age at glasses prescription, ROP type and treatment.

A multivariate analysis was performed to investigate the interdependence of SE change in both groups. The standard lenses group had significantly greater mean SE progression at 6 and 12 months compared to the HALT group, even after adjustment in a multiple logistic regression model for age at glasses prescription, ROP type and ROP treatment requirement (*p* < 0.0001). Therefore, these parameters did not affect the efficacy of HALT technology in reducing MOP progression at both timepoints (*p* = 0.947 and *p* = 0.669 for age at prescription; *p* = 0.503 and *p* = 0.433 for ROP type; *p* = 0.503 and *p* = 0.433 for ROP treatment), which appeared to be primarily related to the type of spectacle lenses used (Figure 3).

**Figure 4:**
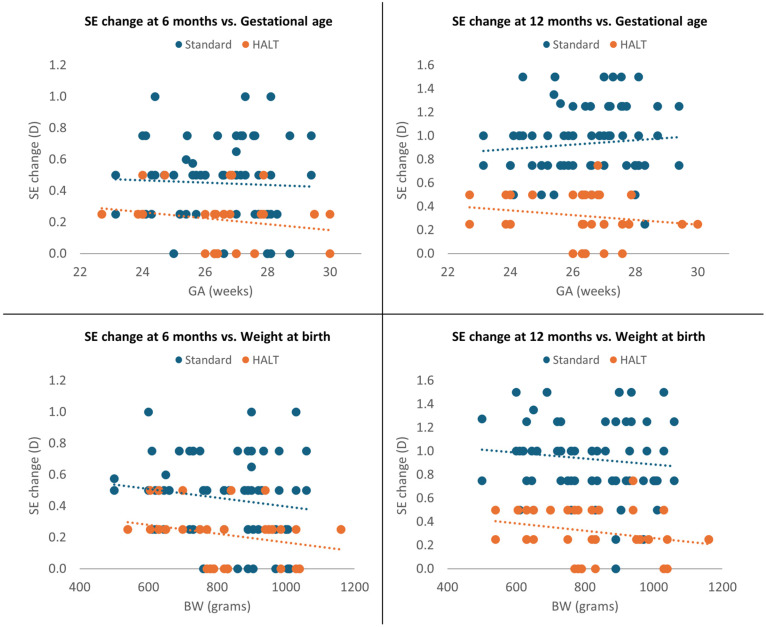
Multivariate analysis at 6 and 12 months adjusted for GA and BW.

Moreover, SE change was significantly greater in the standard lenses group at both 6 and 12 months, even after adjustment for GA and BW (*p* < 0.0001). SE progression was not significantly correlated with GA in both groups at 6 and 12 months (*p* = 0.466 and *p* = 0.107) and with BW at 6 months (*p* = 0.108); however, it was inversely associated with BW at 12 months (*p* = 0.041). Children with higher BW exhibited lower MOP progression at 12 months, as shown in Figure 4.

## 4. Discussion

A recent meta-analysis provided risk estimates for the incidence of myopia-related complications in the general myopic population, concluding that one in three individuals with high myopia is at risk of developing bilateral low vision with age [23]. Although individuals with low and moderate myopia are less likely to develop such severe visual outcomes, they are nevertheless at a significantly increased risk of myopic macular degeneration, retinal detachment, cataract and open-angle glaucoma. Indeed, Haarman et al. reported that low, moderate and high myopia are all associated with increased risk of myopic macular degeneration (OR, 13.57; OR, 72.74; OR, 845.08, respectively), retinal detachment (OR, 3.15; OR, 8.74; OR, 12.62, respectively) and open angle glaucoma (OR, 1.59; OR, 2.92, for low and moderate/high myopia, respectively) [23]. Therefore, considering that the global prevalence of myopia is increasing, and that, according to the World Health Organization (WHO), it is projected to rise from 22% in 2000 to 52% by 2050, awareness of its complications among patients, physicians and policy makers is crucial, and the development of global strategies for the prevention and treatment of myopia progression is considered a priority [24]. Within this context, HALT technology was recently proposed as a possible tool to reduce school-age myopia progression [17,18,19,20].

MOP can be considered a multifactorial condition, as several factors contribute to its development in the formerly preterm population, including low BW, early GA and ROP severity. These factors are considered the main determinants of impaired ocular growth during early life, primarily affecting the anterior segment [25,26]. According to most authors, MOP should be regarded as a distinct clinical entity from pathologic and school-age myopia; however, a progression of SE and AL during school age is also observed in children with MOP [27]. Although MOP is closely associated with ROP and its treatment, prematurely born individuals remain at risk of developing myopic refractive errors even in the absence of ROP. In the multicenter Trial of Cryotherapy for Retinopathy of Prematurity (CRYO-ROP), refractive error data (collected between 3 months and 2 years post-term in more than 1000 patients) demonstrated that the prevalence of myopia, particularly high myopia, was positively correlated with lower BW and increased ROP severity. The risk of developing myopia at the 3-month and 1-year examinations increased by 10% for each 100 g decrease in BW, while for each incremental increase in acute-phase ROP severity, the risk of myopia increased by at least 50–60% up to the 2-year examination. In the same study, the overall prevalence of myopia in individuals born prematurely was 21%, whereas myopia prevalence in severe ROP reached 80%, with high myopia documented in 43% of cases [6]. These findings were further confirmed by the ET-ROP study, which enrolled patients with pre-threshold ROP and reported a prevalence of myopia and high myopia of approximately 65% and 35%, respectively [28]. According to the WHO, an estimated 13.4 million infants were born preterm in 2020 and 152 million were born preterm over the last decade, corresponding to approximately 1 in 10 births worldwide. Taken together, these data underscore the importance of implementing effective strategies for the prevention and treatment of MOP progression in this high-risk population, with the aim of reducing myopia-related complications.

In the present study, we investigated MOP progression in children born prematurely and previously affected by ROP who were corrected with either conventional single-vision lenses or HALT lenses during childhood. Our results demonstrate a significant reduction in MOP progression in children using HALT technology compared with those wearing standard lenses. At baseline, no significant differences in SE or AL were observed between the two groups, both showing mild-to-moderate myopia. However, after 6 months, the HALT group exhibited a significantly lower myopic progression compared with the standard group. Similarly, at 12 months, myopia progression remained significantly greater in the standard lenses group. These findings indicate that HALT lenses provide superior control of MOP progression compared with conventional single-vision lenses. Our findings are consistent with previous research highlighting the efficacy of highly aspherical spectacle lenses in slowing the progression of school-age myopia in various pediatric populations. Studies by Bao et al. and Li et al. reported significantly slower myopia progression in children wearing HALT lenses compared with those using standard single-vision spectacle lenses [17,18,19,20]. The aspheric lenslet design is thought to reduce peripheral hyperopic defocus, which has been identified as a key factor contributing to myopia progression [16].

In the present study, the difference in MOP progression at both 6- and 12-month follow-up remained significant even after adjustment for potential confounding factors, including GA, BW, ROP type, ROP treatment requirements and age at glasses prescription. This finding suggests that the optical characteristics of HALT lenses play a central role in controlling MOP progression in this cohort. Conversely, GA, BW, ROP-related variables and age at spectacle prescription did not significantly influence treatment efficacy. Nevertheless, we observed that lower BW was associated with greater SE progression at 12 months, indicating that children with more severe prematurity remain at higher risk of myopia progression. Importantly, HALT technology appeared to be effective even in this very high-risk subgroup.

In this study, we excluded subjects treated with laser photocoagulation for type 1 ROP, despite their known increased risk of developing MOP compared with patients treated with anti-VEGF agents [7,8,9,10]. This decision was based on the hypothesis that peripheral retinal ablation may reduce or abolish the efficacy of HALT lenses, as the optical defocus generated by the 1021 lenslets is primarily distributed across the peripheral retina [16]. Moreover, the ROP treatment paradigm has shifted in recent years with the widespread adoption of anti-VEGF therapy, rendering laser photocoagulation an increasingly residual treatment.

The major limitations of this study include the retrospective design and the large number of patients excluded due to incomplete refractive data. Although the relatively small sample size may have limited the statistical power to detect subgroup effects (e.g., differences by ROP severity or BW) and may increase the risk of type I error, the magnitude and consistency of the observed differences in both SE progression and AL elongation suggest a clear clinically relevant effect of HALT lenses in this high-risk population. Another limitation of this study is the relatively short follow-up period. Myopia progression in preterm infants is a chronic process that can extend into adolescence and short-term reductions in SE progression or AL elongation do not necessarily guarantee sustained long-term benefits or a reduced lifelong cumulative risk of myopia-associated visual impairment. Nevertheless, this is the first study to suggest that this approach may be beneficial for this high-risk population. Moreover, clinical evidence on HALT technology in school-aged children demonstrates consistent efficacy at 12 months, with outcomes replicated annually in studies that now report up to five years of follow-up [20].

In conclusion, our study demonstrates that HALT lenses represent an effective, non-invasive intervention for slowing MOP progression in children born prematurely and previously affected by ROP. These lenses offer a promising strategy for managing myopia in this vulnerable population and should be considered as part of a targeted myopia control approach. By reducing the rate of MOP progression, HALT lenses may improve long-term visual outcomes and decrease the risk of future myopia-related ocular complications, ultimately enhancing quality of life. Furthermore, this selected high-risk population may serve as a proof of concept supporting the efficacy of HALT technology in the broader myopic population, suggesting that its use in carefully selected high-risk subgroups may reduce the lifelong cumulative risk of visual impairment associated with myopia.

## Figures and Tables

**Table 1:** Clinical and demographics characteristics of the study population in the standard and HALT groups.

Parameter	Groups		*p*-Value
	Standard Lenses	HALT Lenses	
**Age at prescription, years**			0.665
Mean ± SD	6.3 ± 0.8	6.2 ± 0.8	
Range	5.1–7.6	5.0–7.8	
**Sex**			0.808
Male, *N* (%)	40 (52.6)	22 (55.0)	
Female, *N* (%)	36 (47.4)	18 (45.0)	
**GA, weeks**			0.901
Mean ± SD	26.4 ± 1.5	26.3 ± 1.8	
Range	23.1–29.4	22.7–30.0	
**BW, grams**			0.962
Mean ± SD	818.0 ± 141.6	820.0 ± 168.1	
Range	500.0–1060.0	540.0–1160.0	
**ROP type**			0.406
1, *N* (%)	14 (18.4)	10 (25.0)	
2, *N* (%)	62 (81.6)	30 (75.0)	
**Maximum ROP stage**			0.626
1, *N* (%)	38 (50.0)	20 (50.0)	
2, *N* (%)	24 (31.6)	10 (25.0)	
3, *N* (%)	14 (18.4)	10 (25.0)	
**ROP treatment**			0.406
No, *N* (%)	62 (81.6)	30 (75.0)	
Yes, *N* (%)	14 (18.4)	10 (25.0)	

BW: birth weight; GA: gestational age; HALT: Highly Aspherical Lenslet Target; *N*: number; ROP: retinopathy of prematurity; SD: standard deviation.

**Table 2:** SE values at baseline, 6 and 12 months in standard and HALT groups.

Timepoint	SE (D)		SE Progression (D)		*p*-Value
	Standard Lenses	HALT Lenses	Standard Lenses	HALT Lenses	
**Baseline**					0.846
Mean ± SD	−1.98 ± 1.40	−1.91 ± 1.16	–	–	
Range	−0.75 to −5.75	−0.75 to −5.00			
**6 months**					**<0.0001**
Mean ± SD	−2.43 ± 1.43	−2.13 ± 1.17	−0.45 ± 0.26	−0.22 ± 0.17	
Range	−0.75 to −6.00	−0.75 to −5.25	0.00 to −1.00	0.00 to −1.50	
**12 months**					**<0.0001**
Mean ± SD	−2.92 ± 1.48	−2.23 ± 1.18	−0.93 ± 0.34	−0.32 ± 0.20	
Range	−0.75 to −6.75	−0.75 to −5.25	0.00 to −0.50	0.00 to −0.75	

Statistically significant *p*-value in bold. D: diopters; HALT: Highly Aspherical Lenslet Target; SD: standard deviation; SE: spherical equivalent.

**Table 3:** AL values at baseline, 6 and 12 months in standard and HALT groups.

Timepoint	AL (mm)		AL Elongation (mm)		*p*-Value
	Standard Lenses	HALT Lenses	Standard Lenses	HALT Lenses	
**Baseline**					0.840
Mean ± SD	23.65 ± 0.75	23.62 ± 0.72	–	–	
Range	22.45–25.30	22.46–25.21			
**6 months**					**<0.0001**
Mean ± SD	23.88 ± 0.75	23.67 ± 0.72	0.23 ± 0.04	0.05 ± 0.02	
Range	22.67–25.54	22.50–25.25	0.16–0.30	0.00–0.10	
**12 months**					**<0.0001**
Mean ± SD	24.11 ± 0.76	23.74 ± 0.73	0.46 ± 0.09	0.12 ± 0.05	
Range	22.90–25.77	22.58–25.33	0.30–0.60	0.00–0.22	

Statistically significant *p*-value in bold. AL: axial length; HALT: Highly Aspherical Lenslet Target; SD: standard deviation.

## Data Availability

The data presented in this study are available in the article. Eventual additional data are available on request from the corresponding author.

## Abbreviations

The following abbreviations are used in this manuscript:

AL	Axial length
BCVA	Best corrective visual acuity
BW	Birth weight
GA	Gestational age
HALT	Highly Aspherical Lenslet Target
MOP	Myopia of prematurity
ROP	Retinopathy of prematurity
SD	Standard deviation
SE	Spherical equivalent
VEGF	Vascular endothelial growth factor
WHO	World Health Organization
